# HaploDMF: viral haplotype reconstruction from long reads via deep matrix factorization

**DOI:** 10.1093/bioinformatics/btac708

**Published:** 2022-10-29

**Authors:** Dehan Cai, Jiayu Shang, Yanni Sun

**Affiliations:** Department of Electrical Engineering, City University of Hong Kong, Kowloon, Hong Kong SAR, China; Department of Electrical Engineering, City University of Hong Kong, Kowloon, Hong Kong SAR, China; Department of Electrical Engineering, City University of Hong Kong, Kowloon, Hong Kong SAR, China

## Abstract

**Motivation:**

Lacking strict proofreading mechanisms, many RNA viruses can generate progeny with slightly changed genomes. Being able to characterize highly similar genomes (i.e. haplotypes) in one virus population helps study the viruses’ evolution and their interactions with the host/other microbes. High-throughput sequencing data has become the major source for characterizing viral populations. However, the inherent limitation on read length by next-generation sequencing makes complete haplotype reconstruction difficult.

**Results:**

In this work, we present a new tool named HaploDMF that can construct complete haplotypes using third-generation sequencing (TGS) data. HaploDMF utilizes a deep matrix factorization model with an adapted loss function to learn latent features from aligned reads automatically. The latent features are then used to cluster reads of the same haplotype. Unlike existing tools whose performance can be affected by the overlap size between reads, HaploDMF is able to achieve highly robust performance on data with different coverage, haplotype number and error rates. In particular, it can generate more complete haplotypes even when the sequencing coverage drops in the middle. We benchmark HaploDMF against the state-of-the-art tools on simulated and real sequencing TGS data on different viruses. The results show that HaploDMF competes favorably against all others.

**Availability and implementation:**

The source code and the documentation of HaploDMF are available at https://github.com/dhcai21/HaploDMF.

**Supplementary information:**

Supplementary data are available at *Bioinformatics* online.

## 1 Introduction

RNA viruses are pathogens in many infectious diseases. Notable examples include acquired immunodeficiency syndrome (AIDS), severe acute respiratory syndrome (SARS), influenza, coronavirus disease-2019 (COVID-19), etc. Lacking strict proofreading mechanism during replication, many RNA viruses generate progeny with slightly different genomes (haploytpes). Although many haplotypes possess the same biological properties, some of them can exhibit undesirable properties like resistance to drugs and high transmissibility. For instance, it is estimated that the divergence of the surface proteins *env* of HIV can increase by 1–2% every year (Herbeck *et al.*, 2006), posing a great challenge for drug design. Another example is Omircon of SARS-CoV-2, which transmits much faster than other variants (Viana *et al.*, 2022). Given the high genetic divergence of RNA viral haplotypes, a sample can contain multiple haplotypes due to evolution or coinfection (Bull *et al.*, 2012; Ghedin *et al.*, 2011; Rockett *et al.*, 2022). Being able to output the haplotypes and their abundance in a given sample can provide substantive information for studying viruses’ evolution, transmission, fitness, etc.

Thanks to the rapid development of sequencing technologies, we can characterize viral populations through their sequencing data. Next-generation sequencing (NGS) technologies and third-generation sequencing (TGS) technologies play important roles in virus sequencing. Although NGS technologies (e.g. Illumina) can produce sequencing data with high accuracy (error rate lower than 1%) (Salk *et al.*, 2018), its short length of reads (typically <250 bp) makes full-length haplotype reconstruction difficult. In particular, short reads had limited success in distinguishing highly similar haplotypes because of the difficulty of phasing distant single-nucleotide variants (SNVs). In contrast, TGS platforms can generate much longer reads, making full-length haplotype construction feasible. The representative TGS technologies can produce reads with lengths reaching several kbp, covering entire viral genomes (Flint *et al.*, 2021; McNaughton *et al.*, 2019). Many researchers had utilized TGS data for virus sequencing (Bull *et al.*, 2020; Flint *et al.*, 2021; McNaughton *et al.*, 2019). However, TGS data can have a higher per-base error rate (e.g. 10%) than NGS data (Ardui *et al.*, 2018; Goodwin *et al.*, 2015; Salk *et al.*, 2018), which poses a challenge to distinguish bona fide variants from sequencing errors. Although there are error-correction tools, many of them are not designed for data with multiple haplotypes (Koren *et al.*, 2017; Salmela and Rivals, 2014; Salmela *et al.*, 2017).

Long-read assembly programs can be applied to haplotype reconstruction, but many of them (Kolmogorov *et al.*, 2020; Koren *et al.*, 2017; Li, 2016; Ruan and Li, 2020) are not designed for viral populations with highly similar haplotypes. They tend to output contigs from the most dominant one (Cai and Sun, 2022; Luo *et al.*, 2022). Thus, haplotype reconstruction requires a different set of efforts from the generic assembly.

There are some viral haplotype reconstruction pipelines for NGS data (Ahn and Vikalo, 2017; Ahn *et al.*, 2018; Chen *et al.*, 2018; Posada-Céspedes *et al.*, 2021). Many of them require paired-end reads as input and are not suitable to error-prone TGS data. Three recently published tools use long reads to reconstruct haplotypes. RVHaplo (Cai and Sun, 2022) and iGDA (Feng *et al.*, 2021) are two reference-based tools. They first detect sites containing SNVs from the alignment between reads and a reference genome. Then, reads are concatenated to form the final haplotypes according to the shared SNVs. Without using a reference genome, Strainline (Luo *et al.*, 2022) takes the longest reads as templates and aligns other reads against them for generating error-corrected contigs. It outputs final haplotypes by further identifying the overlaps between contigs. These three tools rely on read/contig overlaps for haplotype reconstruction. It is not trivial to determine an optimal cutoff of consistency for reads with different overlap sizes. Using a relaxed threshold helps construct haplotypes of heterogeneous or low coverage, but may mix reads from highly similar haplotypes. On the contrary, setting a stringent threshold may overestimate the haplotypes due to the high sequencing error rate of TGS data. In addition, lacking the prior knowledge of the number of haplotypes further compounds the problem of choosing optimal thresholds. Thus, the performance of existing viral haplotype reconstruction tools for long reads may fluctuate on datasets with different properties.

In this work, we developed a tool named HaploDMF for viral haplotype reconstruction from long reads. HaploDMF utilizes a deep matrix factorization (DMF) model to learn latent features for distinguishing reads from different haplotypes. In order to achieve more robust haplotype reconstruction performance for data with heterogeneous sequencing coverages, we design a new loss function that incorporates the shared SNVs between reads and also the frequency distribution of SNVs. We benchmarked HaploDMF with the state-of-the-art tools on both simulated and real sequencing data. The experimental results demonstrate that HaploDMF has the most robust performance with high accuracy on datasets with different properties.

## 2 Materials and methods

HaploDMF takes long reads and a reference genome as input. It outputs the number of haplotypes, their genomes, and their relative abundance in a given sample. HaploDMF partitions reads into different haplotypes by clustering the learned latent features from reads. The latent features compress the sequence information from original reads such as position and base frequency, so that it can distinguish reads from different haplotypes more effectively. There are two advantages of using latent features for clustering. First, the latent feature learning process uses all the reads and thus the distance computation between two learned latent features tends to be more accurate than raw reads. Second, using the latent features alleviates the difficulty of adjusting the thresholds for reads with different overlap sizes, leading to more robust performance. There are three steps in HaploDMF. In the first step, HaploDMF identifies potential SNV sites, which captures the differences between haplotypes while discarding the common bases between haplotypes. And then it constructs a matrix recording the base frequencies of reads at each SNV site. Second, HaploDMF employs a deep matrix factorization model (Xue *et al.*, 2017) with an augmented loss function to extract latent features for each read based on the frequency matrix. Third, HaploDMF applies a clustering algorithm on the learned latent features and obtains a set of read clusters representing haplotypes. Then, it outputs the haplotypes and their abundance from read clusters.

### 2.1 Frequency matrix construction

**Fig. 1.**
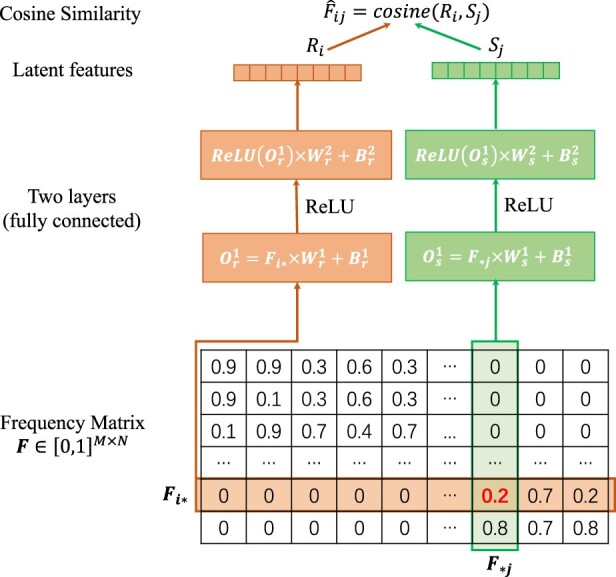
Schematic diagram of the deep matrix factorization model. The frequency matrix (size *M *×* N*) records the base frequency of reads at SNV sites. Each row ($F_{i*}\in R^{1\times M}$) represents the base frequency of a read at all the SNV sites. Each column ($F_{*j}\in R^{1\times N}$) represents the base frequency of all reads at an SNV site. The colored panels in the middle denote the two-layer neural network. In layer *l*, *W^l^* and *B^l^* are the weight and bias of the neural network, and *O^l^* is the output. Subscripts *r* and *c* are used to distinguish the network parameters of learning *R_i_* and *S_j_*, where *R_i_* and *S_j_* are two latent features of the read and the SNV site. ReLU is an activation function. In our design, we set $W_{r}^{1}\in R^{M\times 128},\;W_{r}^{2}\in R^{128\times 32},\;W_{s}^{1}\in R^{N\times 256}$, and $W_{s}^{2}\in R^{256\times 32}$. Thus, *R_i_* and *S_j_* have the same dimension $R^{1\times 32}$. Finally, cosine similarity ${\hat{F}}_{ij}=\mathrm{cosine}(R_{i},S_{j})$ can be applied to the two latent features to approximate the frequency *F_ij_*

Because SNVs indicate the differences between haplotypes, focusing on SNVs helps distinguish reads from different haplotypes. There are different methods for SNV detection (Cai and Sun, 2022; Feng *et al.*, 2021). Users can select an SNV detection tool to generate SNV sites as input to HaploDMF. Here, we use our own method (RVHaplo) based on binomial tests for SNV detection (Cai and Sun, 2022). It outputs a set of SNV sites given the alignment between reads and a reference genome. The recall and precision are usually more than 95% when the sequencing error rate is not high (e.g. 12%). When the dataset has a high sequencing error rate (e.g. 18%), RVHaplo may output some fake SNV sites. For example, the precision and recall of SNV sites on a simulated 5-haplotype dataset with 18% sequencing error rate (Supplementary Fig. S5) are 0.83 and 0.86. Using the learned features from all reads makes HaploDMF more robust against the noise in SNV detection. With the SNV sites, we count the frequencies of four bases at these sites from the alignment and generate a *M *×* N* frequency matrix (see Fig. 1), where *M* and *N* are the number of reads and SNV sites, respectively. *F_ij_* is the base frequency (0$\leq F_{ij}\leq$1) of read *i* at site *j*. For example, if read *i* is aligned to the SNV site *j* with base ‘A’, the frequency of base ‘A’ at the SNV site *j* is assigned to *F_ij_*. If read *i* does not contain a base at site *j*, *F_ij_* = 0.

### 2.2 Deep matrix factorization

Deep matrix factorization (DMF) has been successfully applied in recommender systems for predicting ranking scores (Xue *et al.*, 2017). DMF can learn latent features of users and items, which can be used to predict the ranking score for other items. Because users of similar tastes tend to give similar scores to the same items, their learned latent features are similar too. Similarly, reads from the same haplotype tend to have the same bases at the shared SNV sites and thus show similar frequencies. As a result, the learned latent features from these reads are expected to be close by their cosine distance. Inspired by the recommender system, we build a DMF model to learn latent features representing each read and site (Fig. 1). The optimization goal of DMF in HaploDMF is to learn the latent features of each read and each site so that their cosine distance can predict the observed frequencies. To reduce the impact of sequencing errors during training, we only keep the frequencies of two most dominant bases at each site (see Supplementary Section S4.2 for further details). As a result, each column in the frequency matrix has two non-zero values. Considering that SNV sites with more than two alleles are relatively rare, the frequency matrix maintains nearly all SNV information for training.

Given the frequency vector $F_{i*}$ of read *i* and the frequency vector $F_{*j}$ at site *j*, the DMF model utilizes a pair of fully connected networks to learn latent features *R_i_* and *S_j_* for read *i* and site *j*, respectively (see Fig. 1). Then, a cosine similarity ${\hat{F}}_{ij}=\mathrm{cosine}(R_{i},S_{j})$ between these two latent features is calculated to approximate the observed frequency *F_ij_* ($F_{ij}\neq 0$). By minimizing the square loss of ${\hat{F}}_{ij}$ and *F_ij_*, DMF is able to learn the latent features *R_i_* and *S_j_*. In this process, if read *i* and read i′ originate from the same haplotype, their shared SNV sites (e.g. site j) can lead to highly similar *F_ij_* and $F_{i\prime j}$, thus similar $\mathrm{cosine}(R_{i},S_{j})$ and $\mathrm{cosine}(R_{i\prime },S_{j})$. As a result, the learned features *R_i_* and $R_{i\prime }$ tend to be similar for reads from the same haplotype. Because bases with small frequencies are more likely to be haplotype-specific SNVs, we use a weighted mean square loss (Eqn. (1)) to train the model. It assigns a bigger weight to small *F_ij_*

(1) $$\mathrm{los}s_{ij}^{1}=\frac{1}{F_{ij}}{\left({\hat{F}}_{ij}-F_{ij}\right)}^{2}$$

Optimizing *loss*^1^ enables us to learn the parameters of the fully connected networks in Figure 1. Then, each read *i* can be converted into its latent features *R_i_*, which is used for clustering. Although the reads without overlaps can still have similar latent features through their overlaps with intermediate reads, their latent features may not be that close due to their large distance. Especially, when the distant reads do not have enough intermediate reads to connect them, it becomes more difficult to cluster them into the same group. Thus, we design another loss function as shown in Eqn. (2).

(2) $$\mathrm{los}s_{j}^{2}=\sum_{\begin{array}{c} k=1,\ldots ,N\\ k\neq j \end{array}}\frac{0.5}{N-1}\times (-\mathrm{cosine}(S_{j},S_{k})+1)$$

Minimizing *loss*^2^ enables us to generate similar latent features for distant SNV sites that share similar frequency distributions. As a result, distant reads can have similar latent features if they contain similar frequencies at distant SNV sites. In Eqn. 2, a smaller value of $-\mathrm{cosine}(S_{j},S_{k})$ indicates latent features *S_j_* and *S_k_* are closer. We force the loss function to be non-negative by adding one to the function. And $\frac{0.5}{N-1}$ is to normalize the loss. In the training process, we combine the loss function (1) and (2) to update the parameters in the model as shown in Eqn. (3). Because reads with overlaps still provide more reliable information for clustering, we assign a bigger weight to $\mathrm{los}s_{ij}^{1}$ (0.8 by default).

(3) $$\mathrm{loss}=\sum_{\begin{array}{c} i,j\\ F_{ij}\neq 0 \end{array}}\left(w\cdot \mathrm{los}s_{ij}^{1}+(1-w)\cdot \mathrm{los}s_{j}^{2}\right)$$

### 2.3 Clustering and haplotype reconstruction

With the normalized latent features of reads, we employ a hierarchical clustering algorithm (Ward’s method) (Ward Jr., 1963) to conduct clustering (Pedregosa *et al.*, 2011). And we utilize an elbow method (Thorndike, 1953) to determine the number of haplotypes. The elbow method determines the number of clusters using the within-cluster distance. Here, we use the total number of different bases in clusters as the within-cluster distance. Given a cluster of latent features, we can generate a consensus sequence from the corresponding reads and calculate the number of different bases between reads and the consensus sequences. With the increasing number of clusters, the different bases decrease sharply at the beginning points. At some points, the value will not change significantly because a majority of reads in each cluster are from one haplotype. The turning point is output as the optimal number of clusters. Further details are presented in Supplementary Section S1.

Given the clusters of reads, we use majority vote to output consensus sequences as the constructed haplotypes. And we calculate the abundance of each haplotype based on the number of reads within each cluster. As we have the consensus sequences and their corresponding clusters of reads, we can further polish the consensus sequences with a genome polish tool called Medaka (Oxford Nanopore Technologies, 2018). Our experiments demonstrate that using Medaka further reduces the base error rate of the consensus sequences. HaploDMF will output the polished consensus sequences as the final result.

## 3 Results

**Fig. 2.**
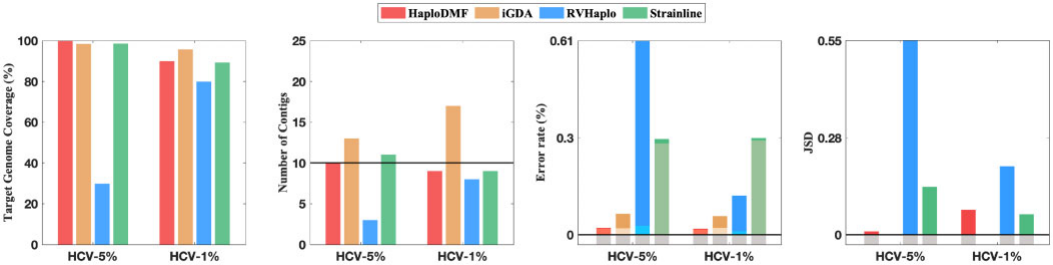
The performance of four tools on two simulated HCV datasets (∼9294 bp, 12% error rate). The black line in the ‘Number of Contigs’ panel indicates the real haplotype number. Two stacking colors (dark and light) and the gray color on each bar in the ‘Error rate (%)’ panel denote the mismatch rate, the indel rate and the zero value, respectively. As iGDA did not output the estimated abundance of haplotypes, it has no results in the ‘JSD’ panel. The ‘N50’ panel is presented in Supplementary Figure S2

**Fig. 3.**
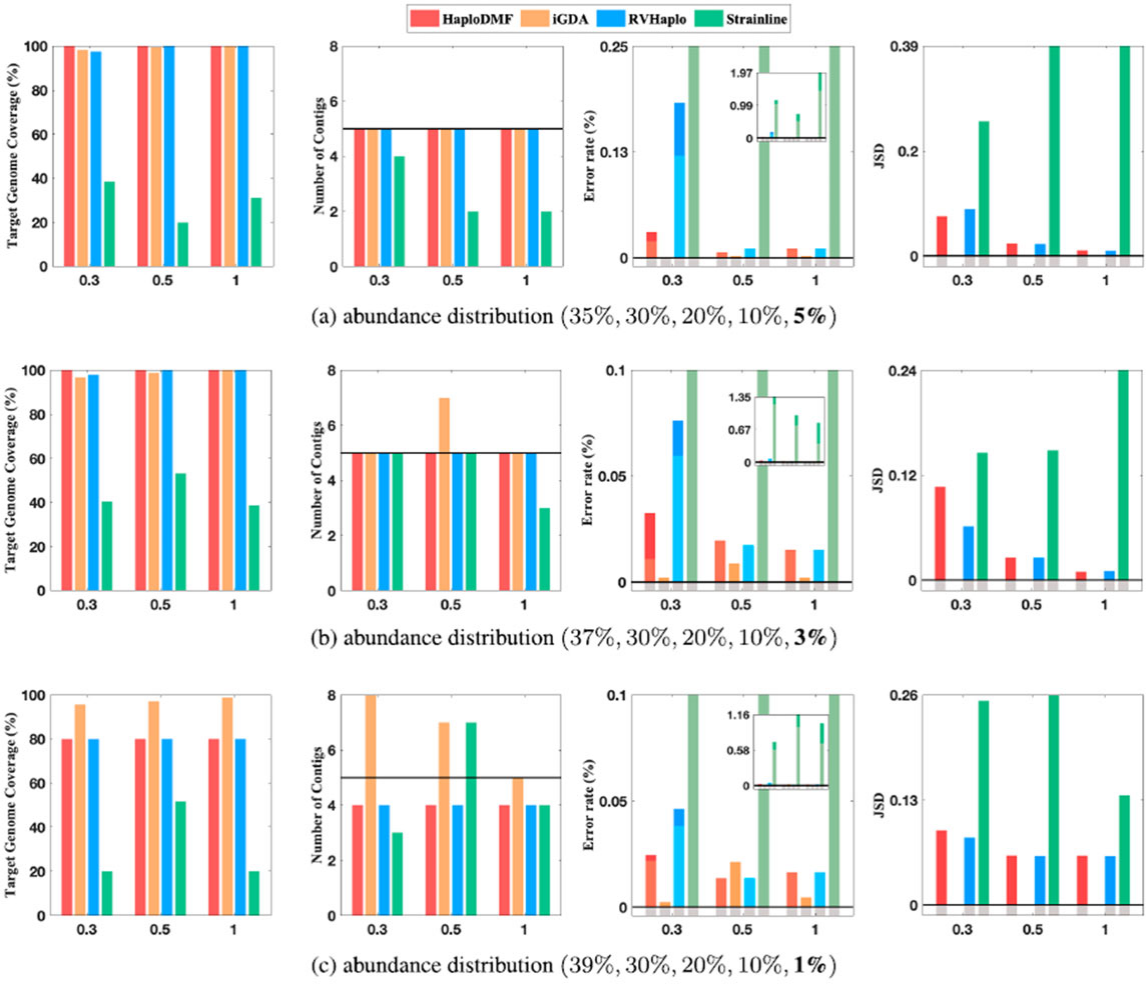
The performance of four tools on simulated HIV datasets (∼9000 bp, 12% error rate) with different divergence settings and abundance distributions. X-axis: the average of pairwise divergences between haplotypes (0.3%, 0.5% and 1%). Complete results including the ‘N50’ panel are presented in Supplementary Figure S4. Other legends are the same as Figure 2. (**a**) abundance distribution (35%, 30%, 20%, 10%, **5%**), (**b**) abundance distribution (37%, 30%, 20%, 10%, **3%**) and (**c**) abundance distribution (39%, 30%, 20%, 10%, **1%**)

**Fig. 4.**
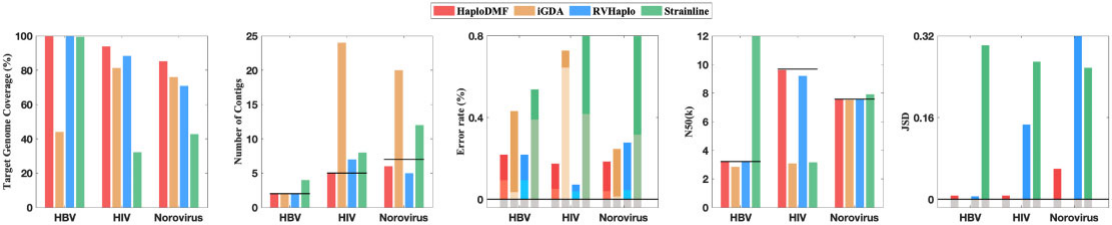
The performance of four tools on real/mock sequencing data: two HBV haplotypes, five HIV haplotypes and seven Norovirus haplotypes. X-axis: three datasets. The black lines in the ‘Number of Contigs’ panel and the ‘N50(k)’ panel indicate the real number of haplotypes and the average length of real haplotypes, respectively. Two stacking colors (dark and light) and the gray color on each bar in the ‘Error rate (%)’ panel denote the mismatch rate, the indel rate and the zero value, respectively. As iGDA did not output the estimated abundance of haplotypes, it has no results in the ‘JSD’ panel

Because different viruses have different replication rates and intra-sample similarities, we conduct experiments on various viruses including hepatitis C virus (HCV), HIV, norovirus, SARS-CoV-2 and a DNA virus, hepatitis B virus (HBV), to evaluate the performance of our method. We tested HaploDMF on both simulated data and real data. In the simulated data experiment, we focus on HIV, HCV and SARS-CoV-2 because HIV and HCV both have high replication rates and tend to form multiple haplotypes in one sample, and SARS-CoV-2 is one of the largest RNA viruses with a genome size of ∼29 903. We first conducted experiments on simulated datasets containing 10 HCV haplotypes (Fig. 2, Supplementary Fig. S2). Then, we tested HaploDMF on simulated datasets containing five simulated HIV haplotypes (Fig. 3, Supplementary Figs S3–S5). To validate HaploDMF on datasets with different properties, we constructed the simulated HIV data with different settings of divergence, abundance, and sequencing error rate. In addition, we used simulated SARS-CoV-2 data to evaluate the performance of HaploDMF on a large virus (Supplementary Section S4.1). Finally, we evaluated HaploDMF on real sequencing data including datasets containing 2 HBV haplotypes, 5 HIV haplotypes and 7 norovirus haplotypes (Fig. 4).

We used seven metrics to evaluate the quality of reconstructed haplotypes and the accuracy of estimated abundance following the previous work. They are target genome coverage, number of contigs (haplotypes), average error rate (summation of the indel rate and the mismatch rate), N50 and Jensen–Shannon divergence (JSD). Of them, target genome coverage evaluates the average percentage of target genomes covered by contigs (haplotypes). Error rate is calculated from the alignments between contigs and the target genomes. JSD measures the similarity between the estimated abundance distribution and the real abundance distribution. It can be calculated by $\mathrm{JSD}(X||Y)=\frac{1}{2}(D(X||Z)+D(Y||Z))$, where *X* and *Y* are two abundance distributions, $Z=\frac{1}{2}(X+Y)$, and *D* is the Kullback–Leibler divergence function. Small value of JSD indicates high similarity between two abundance distributions.

We focus on benchmarking our tool HaploDMF with three recently published haplotype reconstruction tools RVHaplo, iGDA and Strainline. HaploDMF, RVHaplo and iGDA are reference-based tools. They take a reference genome and the alignment between long reads and the reference genome as input. The *de novo* tool Strainline only takes long reads as input. Because HaploDMF and RVHaplo output consensus sequences and read clusters, we are able to apply Medaka to polish their results directly. But iGDA and Strainline need more post-processing steps to apply Medaka. And we found that Medaka did not always improve the results of iGDA and Strainline. Thus, we only showed the results of applying Medaka to HaploDMF and RVHaplo but not to iGDA and Strainline. The details of applying Medaka to the four tools are presented in Supplementary Section S4.3.

### 3.1 Experiments on simulated HCV data

We simulated two datasets for 10 real HCV (genotype 1a) haplotypes (9273–9311 bp) using Badread (Wick, 2019) with an average error rate of 12% and read length of 2500. The accession IDs of the 10 haplotypes are summarized in Supplementary Table S1. These 10 haplotypes were used in Strainline as case studies. The pairwise divergence ranges from 2.8% to 7.4% (Luo *et al.*, 2022). There are 44 700 and 44 783 reads in these two datasets, respectively. According to the smallest abundance of haplotype in the datasets, we used ‘HCV-5%’ and ‘HCV-1%’ to denote the datasets. The HCV-5% dataset has the same abundance distribution setting (5–15%) as Strainline. The smallest and the largest abundance of haplotypes in the HCV-1% dataset are 1% and 19%. The detailed abundance distributions of haplotypes in these two datasets are shown in Table 1 and Supplementary Table S1.

**Table 1.** Abundance distribution of 10 HCV haplotypes

Dataset	Abundance distribution
HCV-5%	**5%**, 6%, 7%, 8%, 9%, 11%, 12%, 13%, 14%, 15%
HCV-1%	**1%**, **3%**, 5%, 8%, 9%, 11%, 12%, 15%, 17%, 19%

We tested the four tools on these two HCV datasets, respectively. For reference-based tools, we used the representative reference genome (NCBI: NC_038882.1) of HCV (genotype 1a) to align reads. Figure 2 shows the results of the four tools. Although iGDA has high target genome coverage on these two datasets, it significantly overestimated the number of haplotypes. RVHaplo, which clusters reads based on identified SNVs, only reconstructed three haplotypes in the HCV-5% dataset and eight haplotypes in the HCV-1% dataset because it grouped many reads from different haplotypes into the same clusters. If we set a more stringent threshold for read clustering, RVHaplo can better distinguish reads from different haplotypes. However, without knowing the number of haplotypes and their relative abundance, it is difficult to adjust the threshold for optimal clustering. With the learned latent features, HaploDMF is able to achieve robust outputs on both datasets. In the HCV-5% dataset, HaploDMF has the highest target coverage and the smallest error rate. It output ten haplotypes with accurate abundance estimation (smaller JSD value) while Strainline generated one more haplotype. In the HCV-1% dataset, HaploDMF has the second-largest target genome coverage and the smallest error rate. HaploDMF and Strainline both reconstructed the nine most dominant haplotypes but missed the 1%-abundance haplotype.

### 3.2 Experiments on simulated HIV data

The HCV experiment with 10 haplotypes allows us to evaluate the performance of the four tools when there are numerous haplotypes. In order to examine the performance of the four tools on datasets with varying divergences, abundance distributions and sequencing error rates, we simulated a series of datasets containing five simulated HIV haplotypes. Given an average divergence (0.3–5%) between haplotypes, we generated five simulated HIV haplotypes from a real HIV haplotype (9181 bp, NCBI: NC_001802.1) by random base mutations. Then, we used Badread to simulate nine datasets with three abundance distribution settings (see Fig. 3) and three average error rates (6%, 12% and 18%). There are ∼43 321 reads in each dataset. And the average read length is ∼2500 bp. Using the real HIV haplotype as the reference for three reference-based tools, we tested the four tools on these datasets and summarized their performance in Figure 3 (12% error rate) and Supplementary Figures S3–S5 (6% and 18% error rate).

In Figure 3, three tools HaploDMF, RVHaplo and iGDA have comparable performance when the smallest abundance of haplotype is 5% or 3% (Fig. 3a and b). They reconstructed five haplotypes with high target genome coverages and small error rates. When the lowest abundance is 1% (Fig. 3c), HaploDMF and RVHaplo only output four haplotypes on datasets with small haplotype divergences (<1%), while iGDA overestimated the number of haplotypes. When the divergence of haplotypes is high (>1%), iGDA and RVHaplo can output the 1% haplotype (Supplementary Fig. S4). The performance of iGDA on the 12%-error-rate datasets is inconsistent with the HCV experiments with the same sequencing error rate. This may be because the lengths and pairwise divergences of haplotypes are the same in the HIV experiments but different in the HCV experiments. We can also observe this in the real data experiments (Fig. 4). Strainline output some contigs (e.g. 4 and 5) on many datasets but the target genome coverages are still small as some contigs are from the same haplotypes. Strainline did not merge them into one contig due to some sequencing errors. Furthermore, if Strainline does not select reads from all haplotypes as templates, it will miss some haplotypes in the final output. The performance of these four tools on the 6%-error-rate datasets (Supplementary Fig. S3) is similar to the 12% error-rate datasets. When the sequencing error rate increases to 18% (Supplementary Fig. S5), the performance of HaploDMF and RVHaplo is still stable. They missed one haplotype on datasets with small haplotype divergences. But iGDA tended to overestimate the number of haplotypes on these high-error-rate datasets.

According to the simulated experiments on HCV and HIV, we observed the following: (i) The performance of HaploDMF is the most robust to the change in haplotypes’ number, divergence, sequencing error rate, and abundance; (ii) iGDA can reconstruct low abundance haplotypes when the sequencing error rate is low. However, it tended to overestimate the number of haplotypes in the following cases: (a) The sequencing error rate is high. (b) The lengths of the genomes and their pairwise divergences vary in the sample. (iii) In most samples, RVHaplo is robust to the change in error rate, divergence, and sequencing error rate. But it needs adjustment of the parameters to achieve optimal results. (iv) Strainline’s performance did not vary significantly across datasets with different properties. It can under- or overestimate the number of haplotypes. In addition, we recorded the running time and memory usage of the four tools on the simulated HIV datasets. The average running time and memory usage of HaploDMF are ∼1 h and ∼19 GB. Further details are presented in the Supplementary file.

### 3.3 Experiments on real/mock sequencing data

We used three real/mock sequencing datasets with increasing difficulty. They are TGS sequencing data from three viruses (including one DNA virus): HBV, HIV and norovirus. The detailed data properties are described in Table 2. *The first dataset* (NCBI: ERR3253560) contains two HBV haplotypes (NCBI: MK321264.1 and MK321265.1) with a high divergence (∼9%). Because reads in this dataset were sequenced with concatemeric amplicons, some reads have much longer lengths than the genome (McNaughton *et al.*, 2019). *De novo* tool (e.g. Strainline) may output haplotypes with extremely long lengths, while reference-based tools can handle the length of haplotypes with this type of data. Because a reference genome with ambiguous bases can fail the running of iGDA, we utilized a HBV genome (NCBI: MT622522.1) without ambiguous bases as the reference for all reference-based tools.

**Table 2.** Overview of three datasets with real/mock sequencing data

Dataset	HBV-2	HIV-5	Norovirus-7
Platform	Nanopore	PacBio	Nanopore
Read number	13 016	15 932	43 492
Median length	1274	1554	2594
Genome size	3212–3215	9535–9719	7513–7618
Divergence (%)	∼9	2.61–8.45	0.95–4.30
Error rate (%)	10.39	2.34	10.19
Abundance (%)	29.1, 70.9	5.8, 14, 17.2, 27.7, 35.3	1, 7, 9.2, 13.8, 18.4, 23.0, 27.6

*Note*: Numbers after the names of viruses denote the number of haplotypes within the datasets.

*The second dataset* (NCBI: SRR961669) is a mock data containing five HIV haplotypes with known genomes (Giallonardo *et al.*, 2014). All the genomes of these five haplotypes can be found in (Giallonardo *et al.*, 2014). And we used the genome of HXB2 as the reference for all reference-based tools because HXB2 is a major circulating haplotype of HIV. Although the error rate of this dataset is small (2.34%), some haplotypes’ sequencing coverages in some regions are very low (Supplementary Fig. S8), impeding the full-length reconstruction of haplotypes. *The third dataset* contains seven haplotypes of norovirus. It was generated by mixing seven single-haplotype dataset. There are many single-haplotype datasets of norovirus by Nanopore sequencing in Flint *et al.* (2021). We chose seven datasets of haplotypes with low pairwise divergence to form this mock dataset. The genomes of haplotypes and datasets can be downloaded from NCBI (Supplementary Table S2). Because the sizes of the seven datasets differ significantly (518–196 525 reads), we randomly sampled reads from each dataset and used the smallest dataset as the 1%-abundance one to create the mock dataset. We only reserved reads that can be aligned to their genomes during the sampling procedure. And we retained reads with lengths between 800 and 8000 because the genome size is about 7.5k. All reference-based tools used a genome of norovirus (NCBI: MW661279.1) from the same project to align reads. The results of applying the four tools to these three datasets are summarized in Figure 4.

In the *HBV experiment*, only HaploDMF and RVHaplo output correct number of haplotypes with the high target genome coverage (∼100%). Although Strainline also has a high target genome coverage (∼100%), it overestimated the number of haplotypes. IGDA generated two haplotypes, but both of them are closer to one of the target genomes. Thus, its target genome coverage on this dataset is small. This may be because iGDA filtered some haplotypes by its stringent filtering conditions. All reference-based tools can generate haplotypes with appropriate lengths, but the *de novo* tool Strainline output extremely long haplotypes as it does not have prior information of the genome length. In addition, HaploDMF and RVHaplo estimated the abundance distribution of haplotypes with high accuracy (HaploDMF: 70% and 30%, RVHaplo: 69.7% and 30.3%). Thus, their JSD values on this dataset are small.

**Fig. 5.**
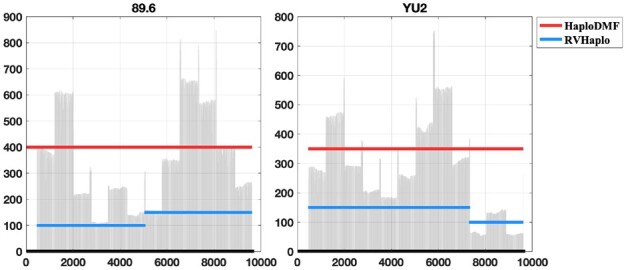
Alignments between contigs and genomes of two haplotypes (89.6 and YU2). The black lines present two haplotypes. The red and blue lines indicate the contigs generated by HaploDMF and RVHaplo, respectively. The grey bars show the sequencing depth of the two haplotypes

In the *HIV experiment*, overlap-based tools RVHaplo, iGDA and Strainline overestimated the number of haplotypes (see Fig. 4). Because the coverages in some regions of haplotypes are small, they failed to concatenate reads into full-length haplotypes, thus outputing many fragmental contigs. For example, RVHaplo output two short contigs for the 89.6 haplotype and the YU2 haplotype. Figure 5 shows the sequencing depth of these two haplotypes and contigs generated by RVHaplo and HaploDMF. The junction regions of two 89.6 contigs and two YU2 contigs generated by RVHaplo have low sequencing coverage. Thus, RVHaplo failed to concatenate two contigs into the full-length haplotype. Because *loss*^2^ helps latent features learn frequency information of distant reads that do not have sufficient overlaps, HaploDMF successfully output two long contigs for 89.6 and YU2. In this dataset, HaploDMF output five long contigs with the highest target genome coverage (∼94%). Without using *loss*^2^, HaploDMF will output short contigs for 89.6 and YU2. And the small JSD value of HaploDMF indicates that the estimated abundance of haplotypes by HaploDMF is close to the ground truth (HaploDMF: 5.5%, 14.1%, 17.6%, 27.2% and 35.6%). Thus, HaploDMF outperforms other tools in this HIV experiment.

**Fig. 6.**
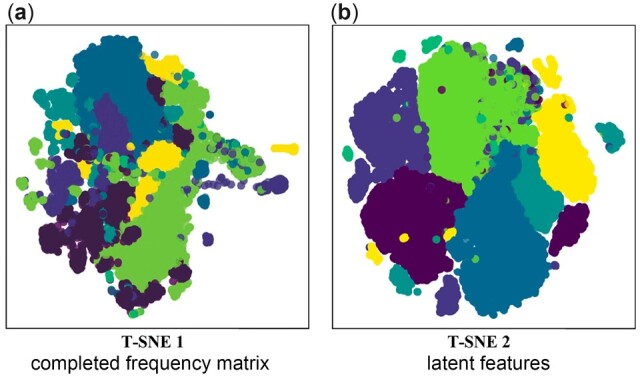
Visualization of the clustering on the norovirus dataset using T-SNE. (**a**) The input data for T-SNE is the completed frequency matrix complemented by HaploDMF. (**b**) The input data for T-SNE are the learned latent features by HaploDMF. Each data point presents the vector of a read. The colors indicate different haplotypes

In the *norovirus experiment*, iGDA and Strainline overestimated the number of haplotypes while HaploDMF and RVHaplo underestimated the number of haplotypes. Compared to other tools, HaploDMF reconstructed 6 haplotypes with the highest target genome coverage and the smallest error rate. It only missed the 1%-abundance haplotype, which is consistent with the simulated experiments. In this experiment, we further investigated the impact of using latent feature vectors on read clustering. As a comparison, we show the visualized clustering performance using the reads’ frequency vectors and the latent feature vectors. In order to cluster the reads’ frequency vectors, we first followed the standard recommender system to predict the ‘missing frequency’ in the frequency matrix (Fig. 1). Then, we used T-SNE (Chan *et al.*, 2019) to map the completed frequency matrix and the latent features into the two-dimensional space. The scatter plot of the mapped data points is shown in Figure 6. Each data point in Figure 6 presents a read and the colors denote different haplotypes. Clear boundaries between color blocks indicate that reads from the same haplotype can be grouped into the same cluster accurately. On the contrary, if the data points with different colors mix together, the clusters will contain reads from multiple haplotypes. In Figure 6, the data points of the latent features are grouped into color blocks clearly, while many data points of the completed frequency matrix mix together with fuzzy boundaries. This comparison further shows the advantage of using latent features for read clustering over using raw frequency vectors from reads.

## 4 Discussion and conclusion

Reconstructing viral haplotypes of a viral population facilitates research on the virus’s evolution, transmission, fitness, etc. TGS technology can generate long sequencing data, which makes full-length haplotype reconstruction more feasible. However, higher per-base error rates in TGS data can lead to overestimation of the haplotypes. Existing error correction tools are not designed for a viral population consisting of multiple highly similar haplotypes. Thus, new methods are still in great need. Existing methods reconstruct haplotypes based on the identity/consistency of read overlaps. They utilize a threshold of base identity in the overlap region to cluster or concatenate reads/contigs. In most cases, the number and genetic similarity of haplotypes in the sample are unknown, making it difficult to decide an appropriate threshold for varying overlap sizes. Thus, the performance of these methods can fluctuate on datasets with different properties (e.g. coverage, sequencing error rate, number of haplotypes, etc.).

In order to address the limitations of clustering reads with small or no overlaps, we developed a tool, HaploDMF, to output genomes of haplotypes and their abundance in samples from TGS data. Instead of relying on the identity of read overlaps directly, HaploDMF utilizes a deep matrix factorization model with a carefully designed loss function to automatically learn latent features from reads. Then, HaploDMF can cluster reads based on the latent features instead of the reads themselves. Thus, regardless of whether reads contain overlaps, HaploDMF can measure their distance with latent features for clustering, which renders HaploDMF more robust performance. In particular, when the dataset has heterogeneous sequencing coverage in some regions, HaploDMF can still reconstruct the full-length haplotypes (as shown in Fig. 5).

HaploDMF is a reference-based tool. Like RVHaplo and iGDA, it requires the user to provide a reference genome of the target virus. If a *de novo* assembly tool can produce a relatively complete contig, the contig can be used as the input. Otherwise, we recommend users to choose a reference genome that is more related to the haplotypes in the sample so that a quality alignment can be obtained for effective SNV detection. In our experiments, we did not purposely choose a closely related genome as a reference. Without assuming much prior knowledge, we choose the representative reference genome from NCBI using the virus name as the keyword in search. Thus, the results can reflect more general usages.

According to our experiments, HaploDMF has the most robust performance on datasets with different properties. On datasets with a typical sequencing error rate (e.g. 10%), HaploDMF can reconstruct haplotypes with at least 0.3% divergence and >1% relative abundance. When the sequencing error rate is high (e.g. 20%), the limits of acceptable abundance and divergence for HaploDMF may slightly increase.

## Supplementary Material

btac708_Supplementary_Data

## Data Availability

The simulation data and the tool of HaploDMF can be downloaded at https://github.com/dhcai21/HaploDMF. The sources of real sequencing data are provided in corresponding sections.
