# Supplementary material for: HaploDMF: viral haplotype reconstruction from long reads via deep matrix factorization
Source: Bioinformatics. 2022 Oct 29;38(24):5360–7. doi: 10.1093/bioinformatics/btac708 (PMC9750122; doi:10.1093/bioinformatics/btac708)
Supplement: btac708_Supplementary_Data [file btac708_supplementary_data.pdf]

Supplementary for HaploDMF: viral Haplotype reconstruction from long reads via Deep Matrix Factorization

Dehan Cai, Jiayu Shang, and Yanni Sun

Contents

1 Determine the number of haplotypes **2**

2 Supplementary tables **2**
2.1 Simulated HCV experiment . . . 2
2.2 Norovirus experiment . . . 3

3 Supplementary figures **3**
3.1 Simulated HCV experiments . . . 3
3.2 Simulated HIV experiments . . . 4
3.3 Sequencing coverage distribution of five real HIV haplotypes . . . 7

4 Supplementary experiments **8**
4.1 Simulated SARS-CoV-2 experiments . . . 8
4.2 Performance comparison of HaploDMF using different frequency matrices . . . 9
4.3 Error rate comparison after applying Medaka . . . 9
4.3.1 The change of error rate after applying Medaka . . . 10
4.3.2 Error rate comparison between consensus sequences and polished sequences for HaploDMF . . . 10

5 Running time and Memory usage **12**

6 Commands of tools **13**
6.1 Versions of tools . . . 13
6.2 Commands . . . 13

1 Determine the number of haplotypes

Suppose we have k clusters of reads generated by a clustering algorithm. For each cluster of read, we output a consensus sequence (only for SNV sites) from reads using majority vote. Then, we calculate the number of different bases (denoted by D_k) between reads and their consensus sequences at the SNV sites. With the increasing value of k, D_k should decrease as the clusters become purer. Once k reaches the real number of haplotypes, the decrease of D_k will become insignificant because a majority of reads in each cluster are from one haplotype. Thus, we calculate the change of D_k with the increasing value of k to determine the number of clusters. If D_{k+1}/D_k > threshold (0.95 by default), we will stop the iteration and output k cluster of reads as the final clusters. However, the number of different bases may not decrease significantly at the beginning iteration when the sequencing error rate is high (See Figure S1). Thus, we will check the change twice (e.g., $D_k/D_{k+1} > 0.95$ and $D_{k+1}/D_{k+2} > 0.90$) to avoid early stopping.

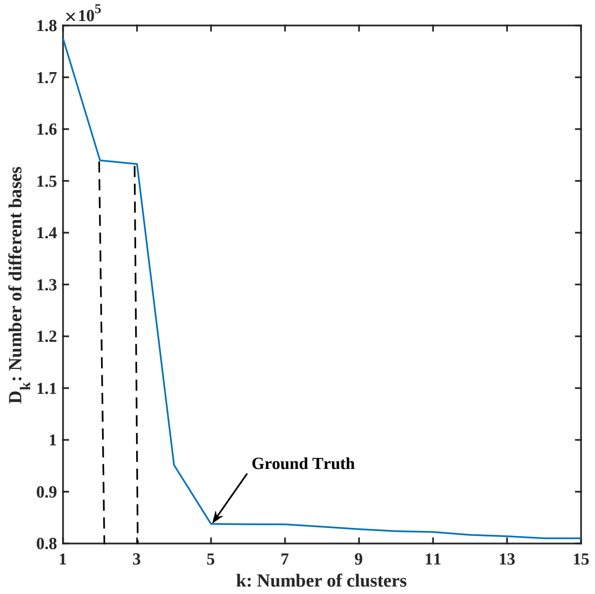

Figure S1: The change of different bases with the increasing number of read clusters on a simulated HIV dataset of 18% sequencing error rate.

2 Supplementary tables

2.1 Simulated HCV experiment

Table S1: Information of 10 HCV haplotypes in the simulated datasets.

Accession ID	Length	Abundance (HCV-5%)	Abundance (HCV-1%)
EU155339.2	9,273	5%	1%
EU155344.2	9,284	6%	3%
EU234065.2	9,273	7%	5%
EU255965.1	9,302	8%	8%
EU255973.1	9,298	9%	9%
EU255980.1	9,296	11%	11%
EU255981.1	9,311	12%	12%
EU255982.1	9,302	13%	15%
EU255983.1	9,297	14%	17%
EU255989.1	9,311	15%	19%

2.2 Norovirus experiment

Table S2: Source of reads and reference genomes for the mixed norovirus dataset.

Dataset	SRA ID	Accession ID	Abundance in the mixed dataset
Norovirus GII strain BMH19-090	SRR15525305	MW661258.1	18.4%
Norovirus GII strain BMH19-092	SRR13951186	MW661259.1	13.8%
Norovirus GII strain BMH19-093	SRR13951185	MW661260.1	23.0%
Norovirus GII strain BMH19-094	SRR13951184	MW661261.1	7%
Norovirus GII strain BMH19-097	SRR13951181	MW661264.1	1%
Norovirus GII strain BMH19-127	SRR13951165	MW661278.1	27.6%
Norovirus GII strain BMH19-137	SRR13951160	MW661283.1	9.2%

3 Supplementary figures

3.1 Simulated HCV experiments

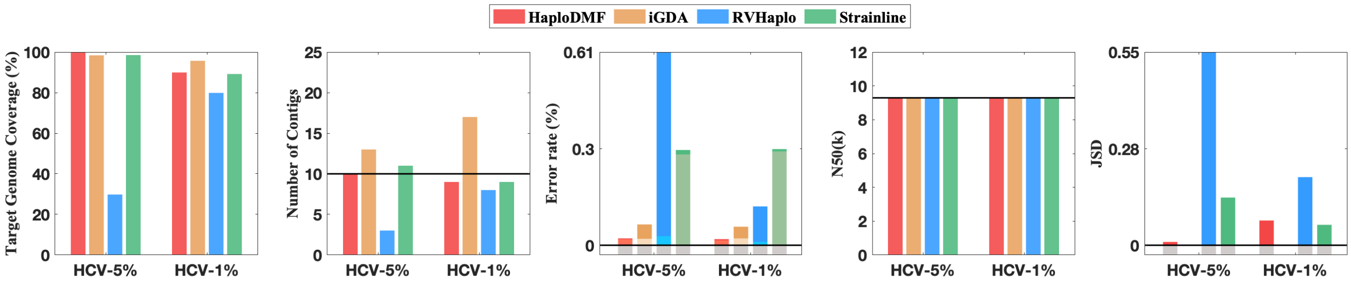

Figure S2: The performance of 4 tools on two simulated 10-HCV datasets ($\sim$ 9294bp, 12% error rate). The black lines in the ‘Number of Contigs’ panel and the ‘N50(k)’ panel indicate the real haplotype number and the average genome length of the real haplotypes, respectively. Two stacking colors (dark and light) and the gray color on each bar in the ‘Error rate(%)’ panel denote the mismatch rate, the indel rate, and the zero value, respectively. As iGDA did not output the estimated abundance of haplotypes, it has no results in the ‘JSD’ panel.

3.2 Simulated HIV experiments

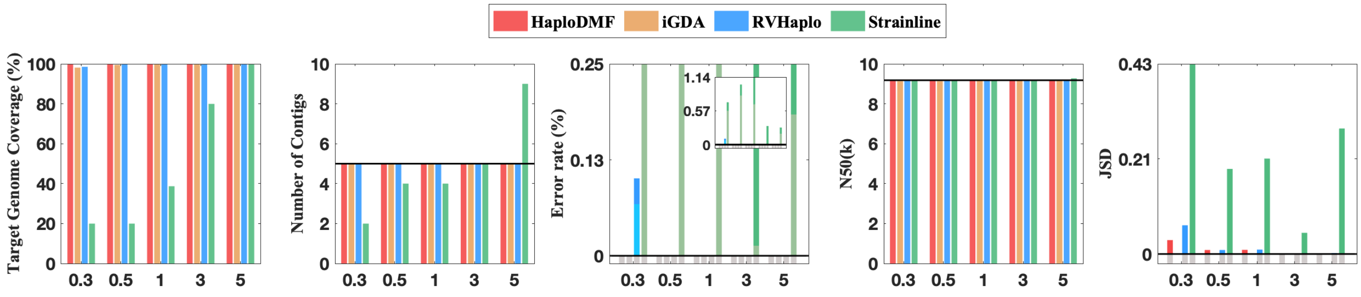

(a) abundance distribution (35%, 30%, 20%, 10%, **5%**)

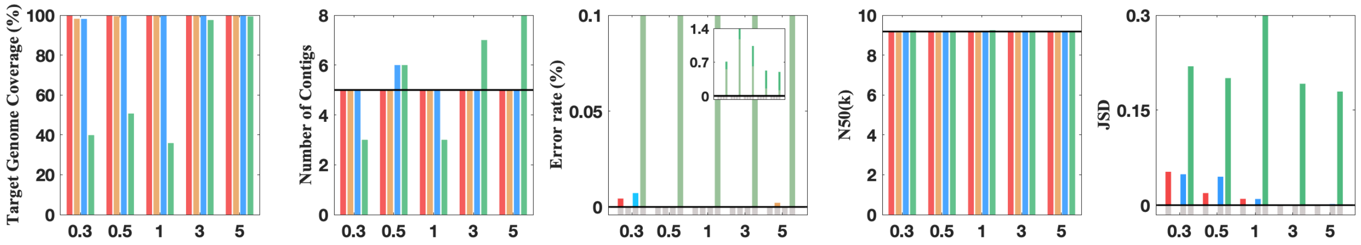

(b) abundance distribution (37%, 30%, 20%, 10%, **3%**)

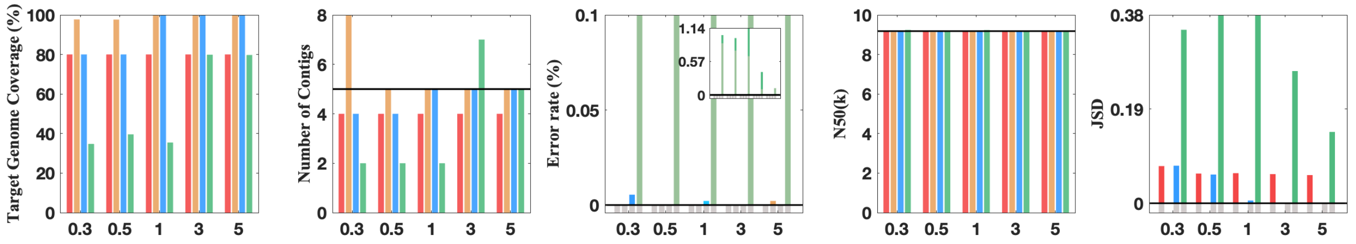

(c) abundance distribution (39%, 30%, 20%, 10%, **1%**)

Figure S3: The performance of 4 tools on simulated 5 HIV datasets (∼9,000bp, 6% error rate) with different divergence settings and abundance distributions. X-axis: the average percentage of pairwise divergences between haplotypes. The black lines in the 'Number of Contigs' panel and the 'N50(k)' panel indicate the real haplotype number and the average genome length of the real haplotypes, respectively. Two colors (dark and light) and the gray color on a bar in the 'Error rate(%)' panel denote the mismatch rate, the indel rate, and the zero value, respectively. As iGDA did not output the estimated abundance of haplotypes, it has no results in the 'JSD' panel.

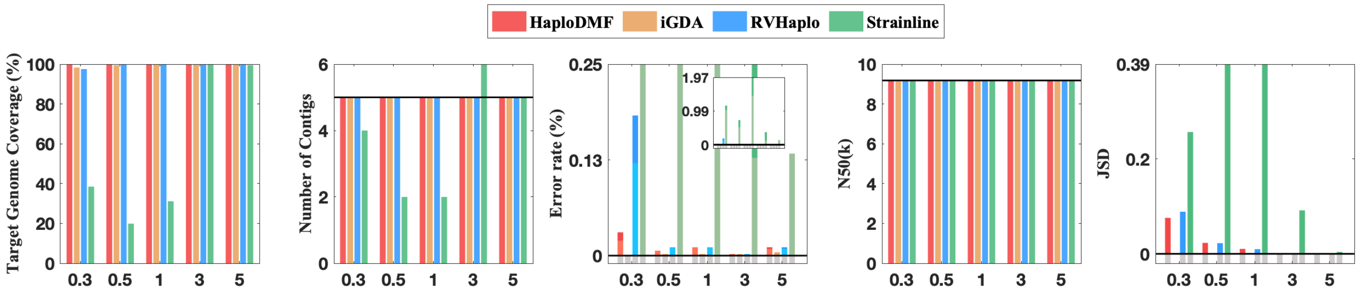

(a) abundance distribution $(35\%, 30\%, 20\%, 10\%, \mathbf{5\%})$

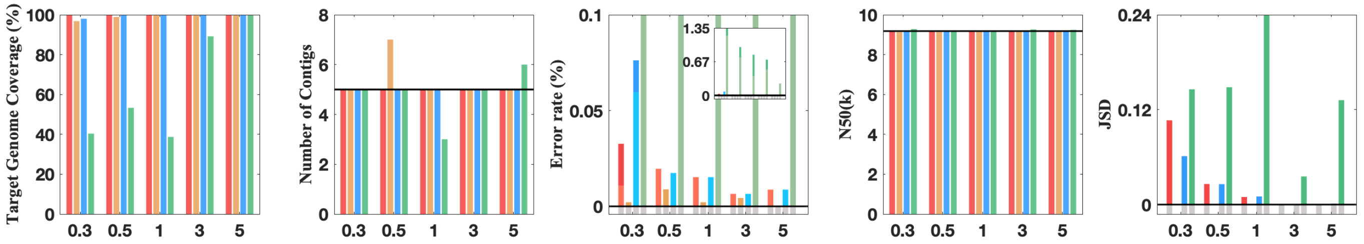

(b) abundance distribution $(37\%, 30\%, 20\%, 10\%, \mathbf{3\%})$

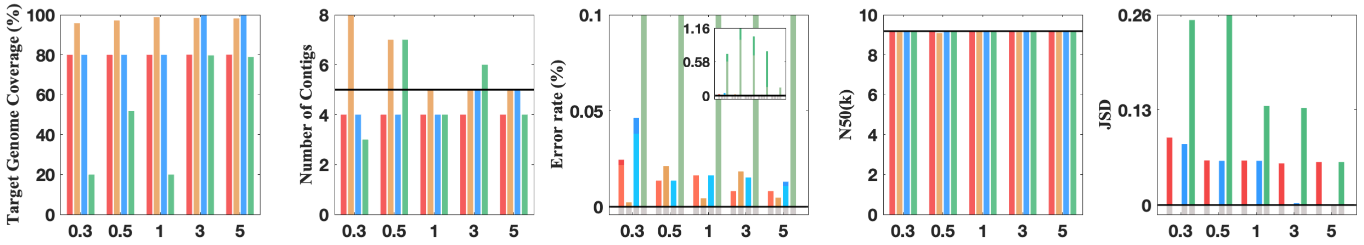

(c) abundance distribution $(39\%, 30\%, 20\%, 10\%, \mathbf{1\%})$

Figure S4: The performance of 4 tools on simulated 5 HIV datasets ($\sim$9,000bp, 12% error rate) with different divergence settings and abundance distributions. X-axis: the average percentage of pairwise divergences between haplotypes. The black lines in the 'Number of Contigs' panel and the 'N50(k)' panel indicate the real haplotype number and the average genome length of the real haplotypes, respectively. Two colors (dark and light) and the gray color on a bar in the 'Error rate(%)' panel denote the mismatch rate, the indel rate, and the zero value, respectively. As iGDA did not output the estimated abundance of haplotypes, it has no results in the 'JSD' panel.

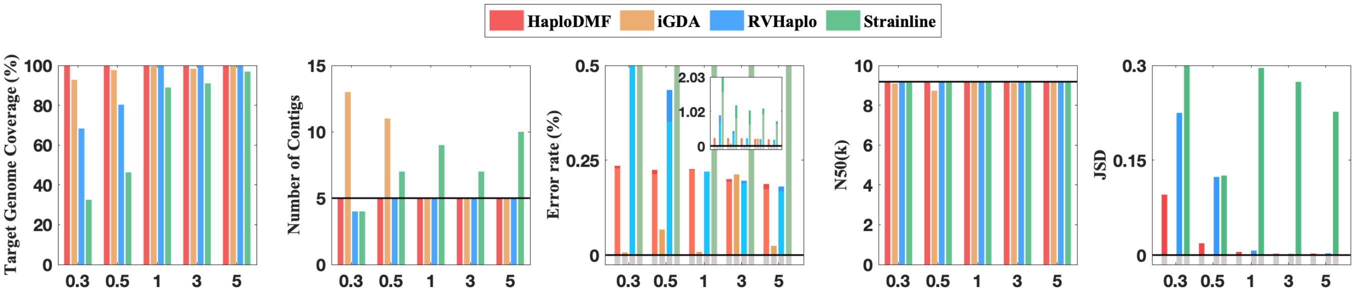

(a) abundance distribution (35%, 30%, 20%, 10%, **5%**)

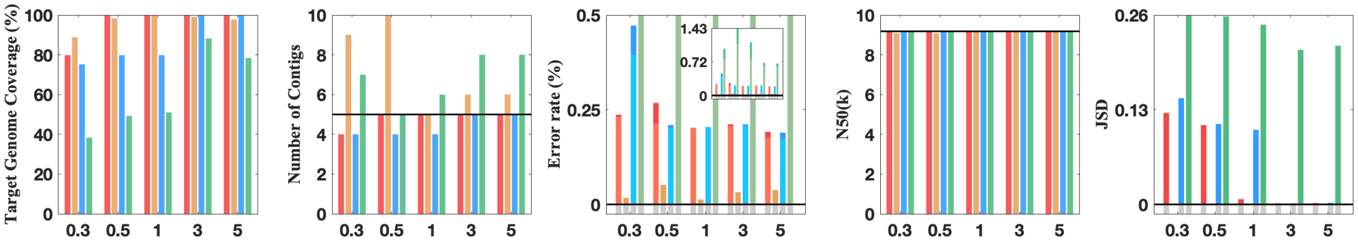

(b) abundance distribution (37%, 30%, 20%, 10%, **3%**)

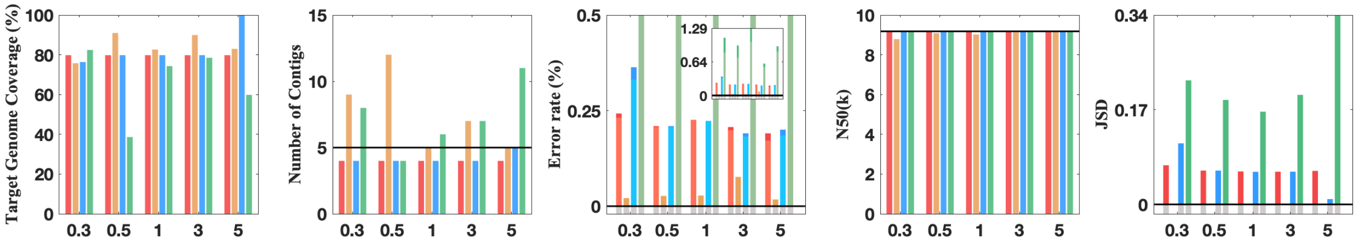

(c) abundance distribution (39%, 30%, 20%, 10%, **1%**)

Figure S5: The performance of 4 tools on simulated 5 HIV datasets (∼9,000bp, 18% error rate) with different divergence settings and abundance distributions. X-axis: the average percentage of pairwise divergences between haplotypes. The black lines in the 'Number of Contigs' panel and the 'N50(k)' panel indicate the real haplotype number and the average genome length of the real haplotypes, respectively. Two colors (dark and light) and the gray color on a bar in the 'Error rate(%)' panel denote the mismatch rate, the indel rate and the zero value, respectively. As iGDA did not output the estimated abundance of haplotypes, it has no results in the 'JSD' panel.

3.3 Sequencing coverage distribution of five real HIV haplotypes

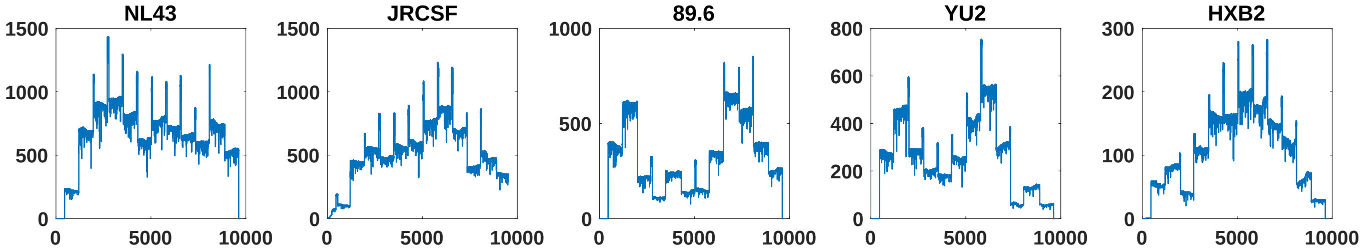

Figure S6: Sequencing coverage distribution of five real HIV haplotypes.

4 Supplementary experiments

4.1 Simulated SARS-CoV-2 experiments

In this section, we evaluated the performance of four tools on the virus with a long genome. Because SARS-CoV-2 is one of the largest RNA viruses with a genome size of $\sim 29,903$, we conducted experiments on it as a case study. There are some variants of SARS-CoV-2 since 2019, e.g., Beta, Delta, and Omicron. It has been reported that different variants can co-infect an individual (e.g., co-infection with Delta and Omicron variants) [1]. Although there are many Nanopore sequencing data of SARS-CoV-2 at NCBI, the haplotype populations within most datasets are unknown. And the true genomes of many datasets are missing, making the evaluation difficult. Thus, instead of testing the four tools on the real sequencing data, we generated three groups of simulated datasets containing two SARS-CoV-2 variants (Omicron: OX006332.1 and Delta: OM739181.1 from NCBI, >99.7% similarity) by Badread with an average error rate of 10% and average read lengths of 10,000, 5,000, and 2,500, respectively. Each group contains three datasets with different abundance distribution settings. We used the abundance of the Delta variant to indicate three datasets: 20%, 10%, and 5%. And there are ∼20,000, ∼40,000 and ∼40,000 reads in datasets from different groups, respectively. The results of applying the four tools to the datasets are presented in Figure S7.

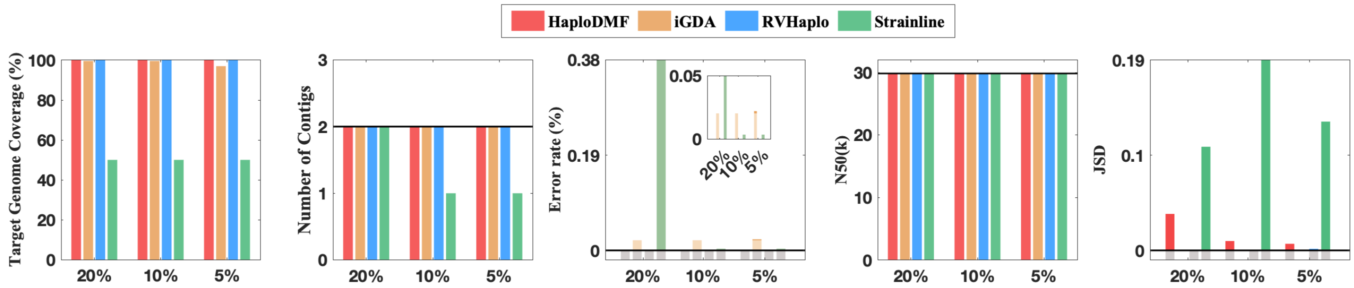

(a) average read length 10,000

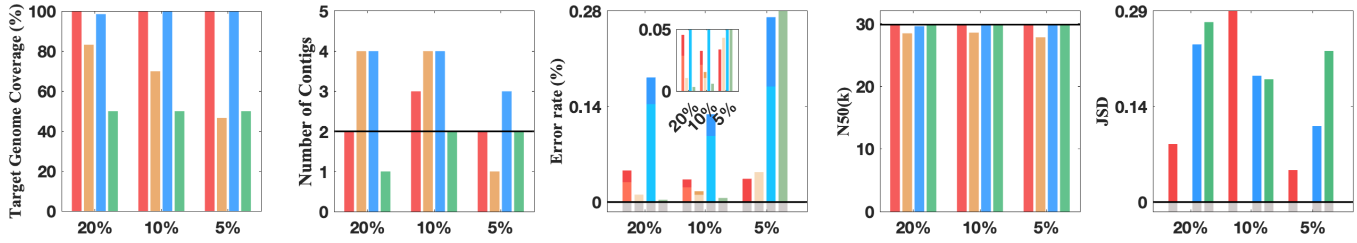

(b) average read length: 5,000

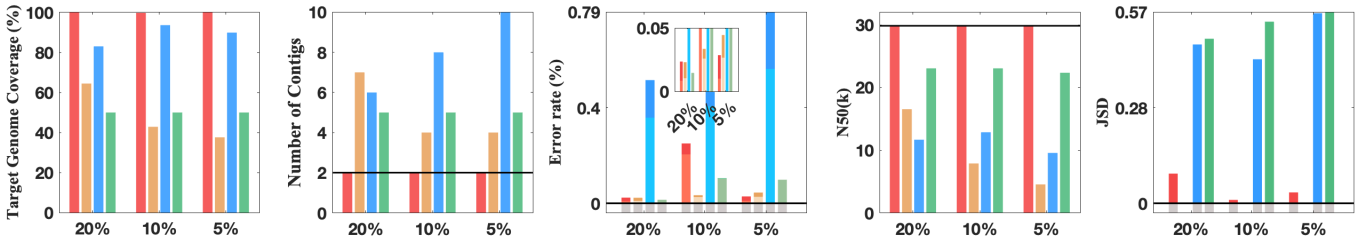

(c) average read length: 2,500

Figure S7: The performance of 4 tools on three groups of simulated SARS-CoV-2 datasets (∼29,903bp, 10% error rate) with different average read lengths. Each group contains three datasets with different abundance distributions. Each dataset contains two haplotypes: Omicron and Delta. X-axis: the abundance of the Delta variant in each dataset. The black lines in the 'Number of Contigs' panel and the 'N50(k)' panel indicate the real haplotype number and the average genome length of the real haplotypes, respectively. Two colors (dark and light) and the gray color on a bar in the 'Error rate(%)' panel denote the mismatch rate, the indel rate and the zero value, respectively. As iGDA did not output the estimated abundance of haplotypes, it has no results in the 'JSD' panel.

Because two haplotypes are highly similar, Strainline only reconstructed the most dominant haplotype on all datasets, thus having ∼50% target genome coverage. When the average read length is 10,000 (Figure S7(a)), HaploDMF, iGDA, and RVHaplo reconstructed the two haplotypes successfully. They all output two haplotypes with high target genome coverages and small error rates. In particular, HaploDMF and RVHaplo output haplotypes with 100% coverage and 100% accuracy. Although HaploDMF did not outperform RVHaplo in estimating the abundance distribution of haplotypes, the estimations are still close to the ground truth, thus having small JSD values. When the average read length becomes shorter (e.g., 5,000 and 2,500), the performance of all tools decreases. For example, RVHaplo and iGDA overestimated the number of haplotypes and generated haplotypes with shorter lengths on datasets with the average read length of 2,500. The error rate of HaploDMF increases with the decrease of

average read length. And its estimated abundance distributions are not as accurate as on the datasets with the average read length of 10,000. But HaploDMF is more robust than other tools on datasets with shorter reads. It reconstructed full-length haplotypes with a high target genome coverage and the correct number of haplotypes even when the average read length (2,500) is about 8% of the genome length.

Our experiments on SARS-CoV-2 demonstrated the feasibility of applying HaploDMF on large RNA viruses. SARS-CoV-2 presents a hard case because of the high similarity between the two haplotypes. In summary, haplotype reconstruction has two limitations for this type of data. First, the large genome and high similarity require long reads to cover at least a couple of SNVs. Shorter reads pose challenges for all tools for large viruses. Second, large viruses require more computational time to identify SNVs. For example, the training time of HaploDMF on a SARS-CoV-2 dataset is about 2 mins using a GPU server. Due to the long genome length, the SNV search part on SARS-CoV-2 takes a long time (∼4h using 8 CPU cores). Thus, applying HaploDMF to large viruses takes longer to finish. To address this challenge, the SNV site detection step supports parallel processing. Using more CPU cores will speed up the process.

4.2 Performance comparison of HaploDMF using different frequency matrices

Given the counts of four bases at each site of a single-haplotype dataset, the most dominant base at each site is usually the correct one while the other three are sequencing errors. Similarly, the top-2 bases at each SNV site in a multiple-haplotype dataset are usually correct if we assume there are only two alleles at each SNV site and the mutation rates of four bases are equal. Thus, we only keep the frequencies of the two most dominant bases at each site to reduce the impact of sequencing errors on training. As a result, each column in the frequency matrix has two non-zero values. Considering that SNV sites with more than two alleles are relatively rare, the frequency matrix maintains nearly all SNV information for training. If we keep all the frequencies at each site to train the model, errors can jeopardize the learning of the latent features. For example, the latent features of two reads from the same haplotype may become less similar caused by the errors. Or, the latent features of two reads from different haplotypes can become similar by chance because of the errors. Thus, clustering based on the latent features can be inaccurate, especially when there are only a few SNVs between highly similar haplotypes.

Taking two types of frequency matrices as input, we tested the performance of HaploDMF on datasets with a high error rate (18%). One frequency matrix (Top-2-Frequency matrix) only reserves frequencies of the two most dominant bases at each site. And the other frequency matrix (All-Frequency matrix) keeps all the frequencies at each site. The results are summarized in Figure S8. When the divergence between haplotypes is high, the performance of HaploDMF using two types of frequency matrices is almost the same. However, when using the All-Frequency matrix for training, the performance of HaploDMF decreases significantly on datasets with small haplotype divergences (0.3% and 5%). It only output one haplotype on these datasets and the error rates are higher than using the Top-2-Frequency matrix. The experimental results demonstrate that removing possible errors before training the network can improve the performance of HaploDMF. Thus, the implementation of HaploDMF uses the Top-2-Frequency matrix to train the network.

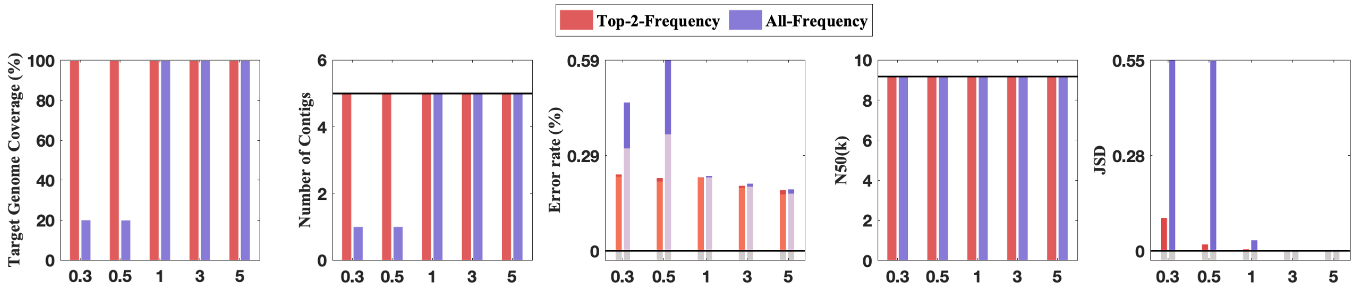

Figure S8: The performance of HaploDMF using different types of frequency matrix on the simulated HIV datasets (∼9,000bp, 18% error rate) used in Figure S5 (a). X-axis: the average percentage of pairwise divergences between haplotypes. Top-2-Frequency: The frequency matrix only reserves the frequencies of two most dominant bases at each SNV site. All-Frequency: The frequency matrix keeps all the frequencies of four bases at each SNV site. The black lines in the 'Number of Contigs' panel and the 'N50(k)' panel indicate the real haplotype number and the average genome length of the real haplotypes, respectively. Two colors (dark and light) and the gray color on a bar in the 'Error rate(%)' panel denote the mismatch rate, the indel rate and the zero value, respectively.

4.3 Error rate comparison after applying Medaka

Medaka is a genome polish tool developed by Oxford Nanopore Technologies. It takes a genome (haplotype) and a set of reads supporting the genome as input and outputs a polished genome. Because HaploDMF and RVHaplo output haplotypes and the corresponding reads for each haplotype, we can easily apply Medaka to polish the original haplotypes. Strainline and iGDA only output the final haplotypes without providing the reads supporting the haplotypes. Thus, more post-processing steps are needed to apply Medaka to the results of Strainline and iGDA.

4.3.1 The change of error rate after applying Medaka

To obtain a set of reads for each haplotype reconstructed by Strainline and iGDA, we realigned all the reads to the haplotypes and clustered reads according to their closest haplotypes (identified by Samtools). Taking the sets of reads and the original haplotypes as input, we can apply Medaka to polish the results of iGDA and Strainline. In order to evaluate the improvement of haplotypes' accuracy after applying Medaka, we calculated the change of error rates between the original and the polished haplotypes on both simulated and real datasets. The results are summarized in Figure. S9, which shows the histograms of the error rate change. Positive and negative values represent increased and decreased error rates after applying Medaka, respectively. In Figure S9, HaploDMF and RVHaplo only have negative values in the histogram, indicating that Medaka reduced the error rates of haplotypes for HaploDMF and RVhaplo on all datasets. However, Medaka did not improve the haplotypes' accuracy for iGDA and Strainline on all datasets. For example, when the sequencing error rate is high (e.g., 18%), the error rate of haplotypes from iGDA increases after applying Medaka. Thus, iGDA and Strainline have both positive and negative values in their panels. It is not trivial to determine the reason. Because there is no clear improvement of applying Medaka to iGDA and Strainline, we keep the original results of iGDA and Strainline in the main text. As HaploDMF and RVHaplo are designed to cluster reads from the same haplotype, reads in a cluster are similar. Thus, using Medaka can improve the quality of haplotypes for HaploDMF and RVHaplo. Although the error rate of the original haplotypes output by HaploDMF is already small (e.g., 0.2% on the norovirus dataset), using Medaka can still help improve the quality of haplotypes for HaploDMF (see Figure S10 and S11).

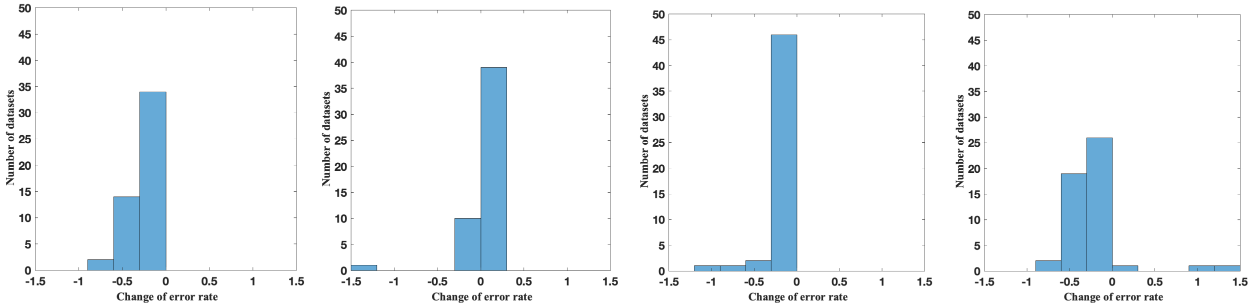

(a) HaploDMF (b) iGDA (c) RVHaplo (d) Strainline

Figure S9: Histogram of the error rate change (percentage) after applying Medaka. X-axis: the change of error rate (after-before). For example, the change of error rate -0.4% is calculated by 0.1%-0.5%. In each panel, positive and negative values represent increased and decreased error rates after applying Medaka, respectively.

4.3.2 Error rate comparison between consensus sequences and polished sequences for HaploDMF

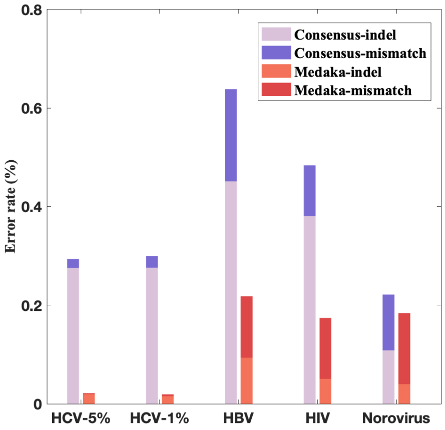

Figure S10: Error rate comparison between consensus sequences and polished sequences for HaploDMF on two simulated HCV datasets and three real/mock sequencing datasets. Consensus-: haplotypes before conducting Medaka-based polisher. Medaka-: haplotypes after applying Medaka. The average difference of error rate before and after applying Medaka is **0.26%**.

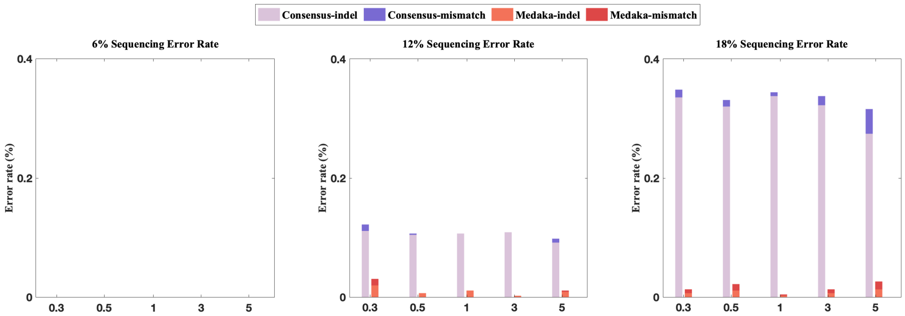

(a) abundance distribution $(35\%, 30\%, 20\%, 10\%, \mathbf{5\%})$

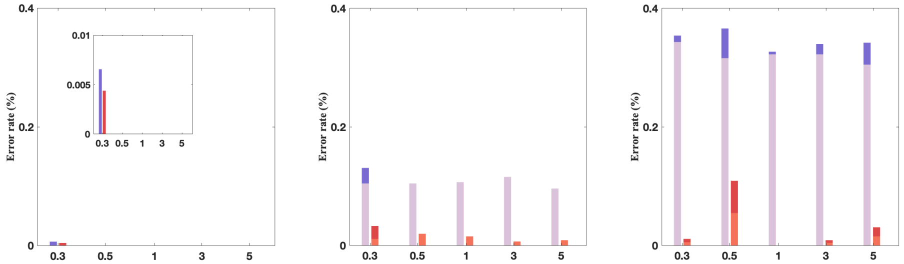

(b) abundance distribution $(37\%, 30\%, 20\%, 10\%, \mathbf{3\%})$

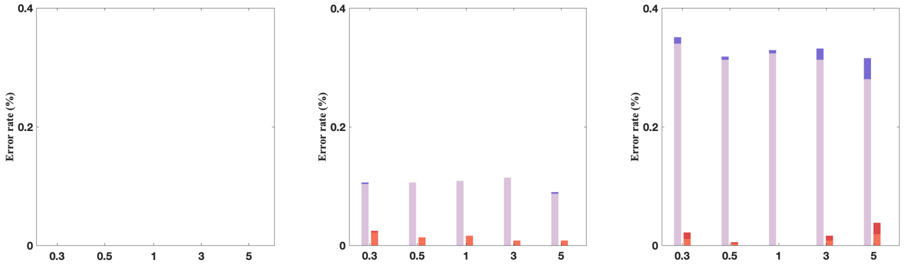

(c) abundance distribution $(39\%, 30\%, 20\%, 10\%, \mathbf{1\%})$

Figure S11: Error rate comparison between consensus sequences and polished sequences for HaploDMF on simulated HIV datasets. X-axis: the average pairwise divergence between haplotypes. Consensus-: haplotypes before conducting Medaka-based polisher. Medaka-: haplotypes after applying Medaka. Blank panels indicate the error rates are zero. The average differences of error rate before and after applying Medaka are **0.094**% and **0.3%** for datasets with 12% and 18% sequencing error rates, respectively.

5 Running time and Memory usage

In the simulated HIV experiments with the 12% sequencing error rate (Figure. S4), we recorded the running time and memory usage of the four tools and summarized the results in Figure S12. Although HaploDMF has the largest running time and memory usage, the running time (∼1h) and memory usage (∼19GB) are acceptable. The SNV detection process and the clustering algorithm contribute most to the running time, while the running time of training the neural network (DMF) ranges from 2 mins to 47 mins depending on the number of SNV sites (0.3%-5% divergence). For the SNV detection process, users can leverage more CPU cores to accelerate the running or users can utilize other SNV detection tools to obtain the SNV sites as input to HaploDMF. For the clustering process, we provide two clustering algorithms (Hierarchical clustering and K-means) in HaploDMF. Our experiments show that they have the same outputs for most experiments and highly similar ones for the rest. If users have datasets with large sizes, they can use K-means to reduce the running time without jeopardizing the clustering accuracy significantly. The high memory usage of HaploDMF is mainly caused by storing the frequency matrix and the learned latent vectors. In addition, using hierarchical clustering algorithm needs more memory than using K-means. User can reduce the memory usage by using the K-means algorithm. In our future work, we will continue to optimize the running time and memory usage of HaploDMF.

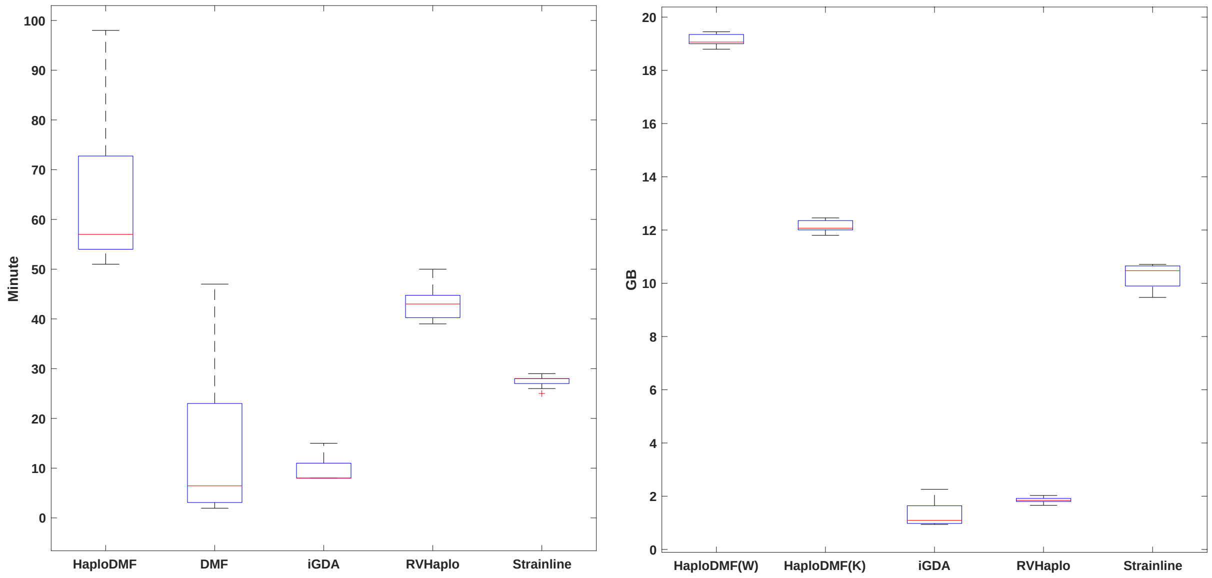

(a) Running time (b) Memory usage

Figure S12: Running time and memory usage comparisons of four haplotype reconstruction tools on the simulated HIV datasets with the 12% sequencing error rate. All the experiments were tested with 8 CPU cores on an HPCC CentOS 7.6.1810 node with 2.70GHz Intel(R) Xeon(R) Gold 6338 CPUs and 160 GB memory. HaploDMF trained the neural network using GPU. 'DMF': the time for training DMF. 'HaploDMF(W)' and 'HaploDMF(K)': HaploDMF with hierarchical clustering (Ward) and K-means, respectively.

6 Commands of tools

6.1 Versions of tools

Table S3: Versions of all used tools

Tool	Version
Minimap2[2]	2.24
iGDA[3]	1.0.1
RVHaplo[4]	v3
Strainline[5]	/
Medaka[6]	1.6.0
Badread[7]	0.2.0

6.2 Commands

Badread

badread simulate –reference haplotype_sequences.fasta –quantity 2000x(HIV)/1000x(HCV) –length 2500,500 –identity 82/88/94,97.5,5 –junk_reads 0 –random_reads 0 –chimeras 0| gzip > simulated.fastq.gz

badread simulate –reference haplotype_sequences.fasta –quantity 3000x/3000x/1500x(SARS-CoV-2) –length 10000/5000/2500,500 –identity 90,97.5,5 –junk_reads 0 –random_reads 0 –chimeras 0| gzip > simulated.fastq.gz

Alignment

minimap2 -a reference.fasta reads.fastq > alignment.sam

HaploDMF

./haplodmf.sh -t 8 -i alignment.sam -r reference.fasta -p haplodmf -o result

iGDA

Nanopore:
igda_align_ont alignment.sam reference.fasta ./alignment/realigned.sam 8
sam2bam ./alignment/realigned.sam 8
igda_pipe_detect -m ont ./alignment/realigned.bam reference.fasta ont_context_effect_read_qv_10_base_qv_10 result_snv
igda_pipe_phase -m ont result_snv reference.fasta result_contigs

PacBio:
igda_align_pb reference.fasta ./alignment/realigned.sam 8
sam2bam ./alignment/realigned.sam 8
igda_pipe_detect -m pb ./alignment/realigned.bam reference.fasta qv10_NCTC_P6_C4 result_snv
igda_pipe_phase -m pb result_snv reference.fasta result_contigs

RVHaplo

./rvhaplo.sh -i alignment.sam -r ref_seq.fasta -o ./result

Strainline

Nanopore:
./Strainline-master/src/strainline.sh –minSeedLen 1000 -i reads.fasta -o result -p ont -t 8

PacBio:

./Strainline-master/src/strainline.sh –minSeedLen 1000 -i reads.fasta -o result -p pb -t 8

Medaka

medaka_consensus -i reads.fastq -d consensus.fasta -o result

References

[1] Rebecca J Rockett, Jenny Draper, Mailie Gall, Eby M Sim, Alicia Arnott, Jessica E Agius, Jessica Johnson-Mackinnon, Winkie Fong, Elena Martinez, Alexander P Drew, et al. Co-infection with sars-cov-2 omicron and delta variants revealed by genomic surveillance. *Nature communications*, 13(1):1–7, 2022.

[2] Heng Li. Minimap2: pairwise alignment for nucleotide sequences. *Bioinformatics*, 34(18):3094–3100, 2018.

[3] Zhixing Feng, Jose C Clemente, Brandon Wong, and Eric E Schadt. Detecting and phasing minor single-nucleotide variants from long-read sequencing data. *Nature communications*, 12(1):1–13, 2021.

[4] Dehan Cai and Yanni Sun. Reconstructing viral haplotypes using long reads. *Bioinformatics*, 38(8):2127–2134, 2022.

[5] Xiao Luo, Xiongbin Kang, and Alexander Schönhuth. Strainline: full-length de novo viral haplotype reconstruction from noisy long reads. *Genome Biology*, 23(1):1–27, 2022.

[6] Oxford Nanopore Technologies. GitHub - nanoporetech/medaka: Sequence correction provided by ONT Research. *GitHub*, `github.com/nanoporetech/medaka`, 2018.

[7] Ryan R Wick. Badread: simulation of error-prone long reads. *Journal of Open Source Software*, 4(36):1316, 2019.
